# Perceptions and Reactions with Regard to Pneumonic Plague

**DOI:** 10.3201/eid1601.081604

**Published:** 2010-01

**Authors:** G. James Rubin, Richard Amlôt, M. Brooke Rogers, Ian Hall, Steve Leach, John Simpson, Simon Wessely

**Affiliations:** King’s College London, London, UK (G.J. Rubin, M.B. Rogers, S. Wessely); Health Protection Agency, Salisbury, UK (R. Amlôt, S. Leach, J. Simpson); Health Protection Agency, Wilts, UK (I. Hall)

**Keywords:** Bioterrorism and preparedness, communication, health behavior, patient compliance, plague, public health, bacteria, respiratory infections, dispatch

## Abstract

We assessed perceptions and likely reactions of 1,005 UK adults to a hypothetical terrorist attack involving pneumonic plague. Likely compliance with official recommendations ranged from good (98% would take antimicrobial drugs) to poor (76% would visit a treatment center). Perceptions about plague were associated with these intentions.

*Yersinia pestis*, the bacterium that causes plague, is a high-priority bioterrorism agent (*1*). The pneumonic form of plague is of particular concern because it can be transmitted from person to person and is fatal if untreated (*2*). However, interventions such as isolating case-patients, identifying contacts, and providing prophylactic antimicrobial drugs may halt the spread of an outbreak (*3*,*4*). The success of such interventions relies on public cooperation, which should not be taken for granted (*5*). Indeed, various commentators have suggested that future plague outbreaks could result in widespread panic (*2*), mass public fear and civil disruption (*1*), and rioting (*6*).

We used a telephone survey of a sample of the adult population of Great Britain to assess their intended behavioral responses in the event of an outbreak of pneumonic plague. We also assessed their perceptions of pneumonic plague and tested whether perceptions were associated with intentions.

## The Study

During September 14–24, 2007, a UK market research company, Ipsos MORI, conducted a random-digit–dial telephone survey. Members of the British population >16 years of age were selected by using proportional quota sampling to ensure that the eventual sample of 1,005 participants was representative of the British public (*7*). King's College London’s Research Ethics Committee approved the study.

The full interview (including several questions not analyzed for this article) and results are in Technical Appendix 1. The survey was conducted in 4 stages. In stage 1, we asked 7 questions concerning perceptions about pneumonic plague. In stage 2, we asked participants to imagine that 3 persons from their area had received a diagnosis of pneumonic plague. To test whether the origin of an outbreak affects responses, 502 participants were also told that police suspected bioterrorism. This manipulation had no effect on most responses. In stage 3, we informed participants that it was now several days later, that the source of the outbreak had been discovered to be a container deliberately hidden at a train station, and that >100 persons had received a diagnosis of plague. In stage 4, we told participants about a specific public health strategy that was being introduced. We informed 502 randomly selected participants about the setting up of mass treatment centers for persons who had been at the train station and told the other 503 that persons who had been at the train station were being asked to stay home for 7 days and to phone a help line if symptoms developed.

In stages 2 and 3, we asked participants whether they intended to undertake specific spontaneous precautionary behavior (questions 12–19 in Technical Appendix 1). An extra item in stage 2 asked whether participants would be willing to take prophylactic antimicrobial drugs if asked to (question 25 in Technical Appendix 1). In stage 4, we asked participants how likely they would be to comply with advice relating to the public health interventions (questions 41–46 in Technical Appendix 1). Before analysis, all responses were weighted according to participant age, sex, work status, region, and social grade.

As expected, precautionary behavior was more likely to be taken in the stage 3 scenario (Tables 1, 2). In terms of likely compliance with official recommendations, 983 (97.8%) participants reported being very or fairly likely to take antimicrobial drugs if asked to. When asked to imagine that they had been to the affected train station, 379 (75.5%) participants reported that they would visit the treatment center immediately if asymptomatic; slightly fewer (331, 65.9%) reported that they would go immediately if they also had influenza-like symptoms. This decrease appeared to be because participants reported that they would likely first consult a primary care physician, hospital, or medical helpline if they had symptoms. In addition, 88 (9.2%) reported being likely to visit the center even if they had not been at the train station, and 141 (28.1%) said that they were likely to visit if they had not been at the train station but had developed influenza-like symptoms. For participants who had been advised to stay home, 459 (91.3%) reported that they would be likely to comply.

**Table 1:** Perceptions of and precautionary behavioral responses to a hypothetical pneumonic plague outbreak affecting 3 persons, United Kingdom, September 2007*

Predictor	Variable level, no. responses			Association, adjusted odds ratio (95% confidence interval)				
	Very or fairly likely	Not very or not at all likely (reference)		Stock up on food, n = 673 (67.2%)†	Leave the area, n = 132 (13.3%)†	Avoid others, n = 746 (74.2%)†	Seek medical advice, n = 667 (66.4%)†	Try to obtain antimicrobial drugs, n = 591 (59.4%)†
If someone catches pneumonic plague, they would feel unwell within 24 h								
	690	149		**1.7 (1.2–2.5)**	1.3 (0.7–2.3)	1.4 (0.9–2.1)	**1.6 (1.2–2.4)‡**	**1.7 (1.2–2.5)**
There have been cases of pneumonic plague in Britain in the past 10 y								
	228	687		0.9 (0.6–1.2)	0.8 (0.5–1.3)	0.8 (0.6–1.2)	1.1 (0.8–1.6)	0.8 (0.6–1.1)
If you come within 6 feet of someone who had pneumonic plague and was clearly ill, you would probably catch the disease								
	735	237		**2.8 (2.0–3.8)**	1.4 (0.8–2.3)	**2.1 (1.5–2.9)**	**2.1 (1.5–2.9)**	**1.9 (1.4–2.6)**
If you come within 6 feet of someone who had pneumonic plague but who had not yet developed any signs of illness, you would probably catch the disease								
	623	333		**2.0 (1.5–2.7)**	**2.0 (1.3–3.2)**	**2.2 (1.6–3.0)**	**2.1 (1.5–2.8)**	**1.9 (1.4–2.5)**
Unless they receive immediate treatment, then most people who catch pneumonic plague will die from it								
	767	169		**1.9 (1.3–2.7)**	**2.7 (1.4–5.4)**	**1.9 (1.3–2.7)**	**2.1 (1.4–3.0)**	**1.6 (1.1–2.3)**
If antibiotics are administered immediately after a person has been infected, they would probably survive								
	880	69		**0.5 (0.3–1.0)**	0.5 (0.3–1.0)	0.7 (0.4–1.4)	0.7 (0.4–1.3)	0.7 (0.4–1.3)
If someone with plague has been in a room, how long would it take after they leave before it is safe to enter the room?								
<1 d	372			**0.6 (0.4–0.8)**	**0.4 (0.2–0.7)**	**0.4 (0.2–0.6)**	**0.6 (0.4–0.8)**	**0.6 (0.4–0.9)**
1–2 d	226			1.0 (0.6–1.5)	**0.6 (0.4–1.0)**	**0.5 (0.3–0.8)**	0.8 (0.5–1.2)	1.0 (0.7–1.5)
>3 d	237			Reference	Reference	Reference	Reference	Reference

*All odds ratios adjusted for home ownership, ethnicity, sex, age, working status, number of years of education, and social grade. Survey stage 2. **Boldface** indicates significance (p<0.05). †Very or fairly likely to perform that behavior.

**Table 2:** Perceptions of and precautionary behavioral responses to a hypothetical pneumonic plague outbreak affecting >100 persons, United Kingdom, September 2007*

Predictor	Variable level, no. responses			Association, adjusted odds ratio (95% confidence interval)				
	Very or fairly likely	Not very or not at all likely (reference)		Stock up on food, n = 798 (79.8%)†	Leave the area, n = 223 (22.4%)†	Avoid others, n = 850 (84.6%)†	Seek medical advice, n = 792 (79.4%)†	Try to obtain antimicrobial drugs, n = 724 (72.5%)†
If someone catches pneumonic plague, they would feel unwell within 24 h								
	690	149		**1.8 (1.1–2.7)**	1.6 (1.0–2.5)	1.3 (0.8–2.1)	1.5 (1.0–2.4)	**1.9 (1.2–2.8)**
There have been cases of pneumonic plague in Britain in the past 10 y								
	228	687		1.1 (0.8–1.7)	0.9 (0.6–1.4)	1.0 (0.6–1.5)	1.4 (0.9–2.1)	0.8 (0.5–1.1)
If you come within 6 feet of someone who had pneumonic plague and was clearly ill, you would probably catch the disease								
	735	237		**2.5 (1.8–3.6)**	**1.8 (1.2–2.7)**	**1.8 (1.2–2.6)**	**2.2 (1.5–3.1)**	**2.0 (1.4–2.8)**
If you come within 6 feet of someone who had pneumonic plague but who had not yet developed any signs of illness, you would probably catch the disease								
	623	333		**2.2 (1.6–3.2)**	**1.4 (1.0–2.0)**	**1.5 (1.0–2.1)**	**2.0 (1.5–2.9)**	**1.5 (1.1–2.1)**
Unless they receive immediate treatment, then most people who catch pneumonic plague will die from it								
	767	169		**2.1 (1.4–3.1)**	**2.8 (1.7–4.7)**	**1.8 (1.1–2.8)**	**2.3 (1.5–3.4)**	**2.2 (1.5–3.2)**
If antibiotics are administered immediately after a person has been infected, they would probably survive								
	880	69		0.6 (0.3–1.2)	0.7 (0.4–1.3)	1.1 (0.6–2.2)	0.4 (0.2–1.0)	1.0 (0.6–1.8)
If someone with plague has been in a room, how long would it take after they leave before it is safe to enter the room?								
<1 d	372			**0.6 (0.4–0.9)**	**0.5 (0.4–0.8)**	**0.4 (0.2–0.7)**	**0.6 (0.4–0.9)**	**0.6 (0.4–0.9)**
1–2 d	226			1.3 (0.7–2.1)	**0.6 (0.4–0.9)**	0.6 (0.3–1.0)	0.7 (0.4–1.2)	1.0 (0.6–1.6)
>3 d	237			Reference	Reference	Reference	Reference	Reference

*All odds ratios adjusted for home ownership, ethnicity, sex, age, working status, number of years in education, social grade, number of people at home and parental status. Survey stage 3. **Boldface** indicates significance (p<0.05). †Very or fairly likely to perform that behavior.

The associations between demographic variables and precautionary behavior are shown in Tables 1 and 2 of Technical Appendix 2). Associations between perceptions and precautionary behavior were adjusted for relevant demographic variables (Tables 1, 2). In general, participants who perceived pneumonic plague to be more severe, easier to catch, or more persistent in the environment were more likely to engage in precautionary behavior (Tables 1, 2). Table 3 in Technical Appendix 2 shows the associations between demographic characteristics and the likelihood of not complying with public health recommendations. Table 4 in Technical Appendix 2 shows the equivalent associations for perceptions about plague, after adjustment for relevant demographic variables. Only unnecessary visits to a treatment center were associated with perceptions; participants who felt that there had been cases of plague in the United Kingdom in the past 10 years (odds ratio [OR] 2.3, 95% confidence interval [CI] 1.3–4.0) or who felt that asymptomatic persons might be contagious (OR 2.8, 95% CI 1.5–5.3) were more likely to report that they would visit the treatment center if they had not been to the affected train station, and participants who believed that antimicrobial drugs are an effective treatment for plague were less likely to report that they would visit (OR 0.3, 95% CI 0.2–0.6).

## Conclusions

Our survey indicates that should an outbreak of pneumonic plague occur, the inclination of the British public would be to adopt a range of spontaneous precautionary behaviors. Intended compliance with possible public health recommendations ranged from excellent (taking prophylactic antimicrobial drugs) to poor (visiting treatment centers). Some intended behavior we identified might complicate management of an outbreak. In particular, ≈25% of potentially exposed persons would not visit a treatment center, yet ≈10% of unexposed persons would. Given that specific perceptions about pneumonic plague were associated with being likely to engage in precautionary behavior, explicitly, clearly, and repeatedly addressing misperceptions during the early stages of an outbreak might help reduce public anxiety and help with decision making (*8*). However, perceptions showed few associations with willingness to comply with explicit public health advice.

Several caveats should be considered with regard to our methods. First, the large number of statistical tests that we conducted and the wide confidence intervals for some of our results make type 1 and type 2 errors likely. Second, our sample probably underrepresented groups who might be more vulnerable in the context of an outbreak, e.g., those who do not have access to a telephone or do not speak English. Our sample also consisted solely of persons who complied with a request to participate in a survey and who might therefore be more likely to comply with official advice during an outbreak. Our results may therefore overestimate likely compliance during an outbreak. Finally, respondents’ difficulty in predicting how they would react to this hypothetical scenario also creates difficulty in assessing validity of results. We therefore caution readers to treat our results as suggestive of the broad level of compliance and precautionary behavior that might occur during an outbreak of pneumonic plague, not as precise predictions.

## Supplementary Material

Technical Appendix 1 Pneumonic Plague Survey King's College London TOPLINE RESULTS 26 September 2007

Technical Appendix 2 Perceptions and Reactions with Regard to Pneumonic Plague
