# Supplementary material for: Perceptions and Reactions with Regard to Pneumonic Plague
Source: Emerg Infect Dis. 2010 Jan;16(1):120–2. doi: 10.3201/eid1601.081604 (PMC2874346; doi:10.3201/eid1601.081604)
Supplement: Technical Appendix 1 — Pneumonic Plague Survey King's College London TOPLINE RESULTS 26 September 2007 [file 08-1604_Techapp1-s1.pdf]

Ipsos MORI

Pneumonic Plague Survey
King's College London
TOPLINE RESULTS 26 September 2007

- Results are based on sample of 1,005 respondents aged 16+, interviewed by telephone.
- Fieldwork was conducted between 14th – 24th September 2007
- Quotas have been set on age, gender, work status, region and social grade
- Data is weighted to the known profile of the population
- Where figures do not add up to 100, this is due to multiple coding or computer rounding
- An asterisk (*) denotes any value of less then half of one per cent, but more than zero
- Responses are based on all interviews unless otherwise specified
- Where base sizes are smaller than 50, number of responses rather than percentages are used

---

Q1. **How is your health in general? Would you say it was…**

	%
Very good	39
Good	41
Fair	15
Poor	3
Very poor	2
Don't know	*

Q2. **Do you have any long-standing illness, disability or infirmity? By long-standing I mean anything that has troubled you over a period of time, or that is likely to affect you over a period of time.**

	%
Yes	24
No	75
Don't know	*

One of the things we are interested in is how people would cope if a disease appeared. In particular, we are thinking of a disease called pneumonic plague. This is a virus similar to the plagues that occurred in Britain in mediaeval times. It can be lethal, and is passed between people by coughing or sneezing. The best known treatment for plague is to give the patient high doses of antibiotics as quickly as possible.

Q3-Q8 **We would now like to find out what you currently think about pneumonic plague. For each please tell me how likely or unlikely you think each statement is. We do not expect that you will know all the answers, but are just interested in what you have heard about the topic.**

		Very likely %	Fairly likely %	Not very likely %	Not at all likely %	Don't know %
Q3.	**If someone catches pneumonic plague they would feel unwell within 24 hours of catching it**	36	33	12	2	17
Q4.	**There have been cases of pneumonic plague in Britain within the past 10 years**	7	16	39	29	9
Q5.	**If you were to come within six feet of somebody who had pneumonic plague and who was clearly ill, you would probably catch the disease from them**	34	39	20	4	3
Q6.	**If you were to come within six feet of somebody who had pneumonic plague but who had not yet developed any signs of illness, you would probably catch the disease from them**	21	41	28	6	5
Q7.	**Unless they receive immediate treatment, then most people who catch pneumonic plague will die from it.**	42	34	15	2	7
Q8.	**If antibiotics were administered immediately after a person had been infected with pneumonic plague, they would probably survive**	41	47	6	1	5

Q9. **If somebody with pneumonic plague has been in a room, after they leave, how long do you think it would take before it is safe for someone else to enter that room and not become ill?**

	%
Immediately / right away	7
Less than 6 hours	14
More than six hours but less than one day	16
1-2 days	23
3-7 days	8
1-2 weeks	7
3-4 weeks	1
Up to 1 month	2
More than a month	3
Never	2
Don't know	17

I'd now like to present a hypothetical situation in which there has been an outbreak of pneumonic plague in Britain. Please imagine that you hear on the news that three people from your local area – within a few miles of you – have been diagnosed as having pneumonic plague. They had been taken to hospital suffering from flu-like symptoms.

Experts are trying to identify the source of the outbreak. They are also trying to trace anybody who has been in close contact with the affected people in order to give them antibiotics as a precaution. They are advising that other people should continue on with their daily lives as normal and that anybody who is concerned about their own health should call NHS Direct, the NHS' telephone helpline.

SPLIT SAMPLE: VERSION A (n=492) AND VERSION B (n=513)

FOR ALL IN VERSION A READ OUT: **Police say they are concerned that the outbreak may be the work of terrorists.**

Q10. **If this situation occurred, how worried, if at all, would you be about catching pneumonic plague yourself?**

	Version A<br>%	Version B<br>%
Very worried	33	23
Fairly worried	36	35
Not very worried	22	33
Not at all worried	7	9
Don't know	1	0

Q11. **If this situation occurred, how worried, if at all, would you be about your close family or loved ones catching pneumonic plague?**

	Version A<br>%	Version B<br>%
Very worried	43	41
Fairly worried	34	34
Not very worried	19	18
Not at all worried	4	6
Don't know	1	1

VERSION A

Q12.-Q19. **And how likely, if at all, would you be to do each of the following actions I am going to read out?**

	**VERSION A**	Very likely<br>%	Fairly likely<br>%	Not very likely<br>%	Not at all likely<br>%	Don't know<br>%	Not applicable (option for Q15 ONLY)<br>%
Q12.	**Make sure your home was stocked up with food and supplies**	49	23	19	8	*	N/A
Q13.	**Carry on as normal**	46	31	15	7	*	N/A
Q14.	**Leave the area**	6	9	38	47	*	N/A
Q15.	**Avoid going to work or college if applicable**	19	12	28	21	3	17
Q16.	**Avoid crowded areas such as public transport, supermarkets or pubs**	44	29	15	11	1	N/A
Q17.	**Avoid leaving your home if at all possible**	21	18	33	27	1	N/A
Q18.	**Seek medical advice**	45	24	20	12	*	N/A
Q19.	**Try to obtain a supply of antibiotics for yourself or your family/household**	39	19	26	14	1	N/A

VERSION B

Q12.-Q19. **And how likely, if at all, would you be to do each of the following actions I am going to read out?**

	VERSION B	Very likely %	Fairly likely %	Not very likely %	Not at all likely %	Don't know %	Not applicable (option for Q15 ONLY) %
Q12.	**Make sure your home was stocked up with food and supplies**	39	22	29	10	*	N/A
Q13.	**Carry on as normal**	51	33	11	5	0	N/A
Q14.	**Leave the area**	6	6	37	49	1	N/A
Q15.	**Avoid going to work or college if applicable**	18	10	26	24	4	18
Q16.	**Avoid crowded areas such as public transport, supermarkets or pubs**	44	26	19	11	1	N/A
Q17.	**Avoid leaving your home if at all possible**	20	16	36	28	*	N/A
Q18.	**Seek medical advice**	46	20	22	13	0	N/A
Q19.	**Try to obtain a supply of antibiotics for yourself or your family/household**	40	19	27	14	1	N/A

Q20. **You said you would be likely to leave the area. Where do you think would you go to?**

Base: All likely to leave the area (VERSION A = 73; VERSION B = 64)

	Version A %	Version B %
Elsewhere in the UK, but within my region of the country	21	13
Elsewhere in the UK, but outside my region of the country	42	49
Elsewhere in Europe but outside the UK	14	11
Outside of Europe	16	16
Don't know	8	11

Q21. **You said you would be likely to see medical advice. Where would you seek medical advice?**

Base: All likely to seek medical advice (VERSION A = 337; VERSION B = 335)

	Version A %	Version B %
GP	73	75
NHS Direct	32	32
Local hospital	19	19
Internet	18	16
Media like newspapers, television or radio	6	6
Medically qualified friend or relative	4	6
Pharmacy	2	3
Another friend or relative (not medically qualified)	*	2
Emergency services	*	1
Other	5	6

Q22. **You said you would be likely to try to obtain a supply of antibiotics. Where would you try to obtain antibiotics from?**

Base: All likely to obtain antibiotics (VERSION A = 289; VERSION B = 300)

	Version A<br>%	Version B<br>%
GP	74	73
Pharmacy	23	26
Local hospital	20	12
Internet	6	7
NHS direct	4	2
Health clinic	2	1
Medically qualified friend or relative	1	2
Another friend or relative (not medically qualified)	1	2
Black market	1	1
Other	4	5
Don't know	1	3

Q23. **Do you have any children under the age of 18 living with you?** PROMPT AS NECESSARY: **And do any of them go to school or nursery?**

	Version A<br>%	Version B<br>%
Yes – but no children are in school or nursery	5	4
Yes – I have one or more children in school or nursery	29	27
No – I have no children / no children living with me	65	69
Refused	0	*

Q24. **How likely or unlikely is it that you would keep your children home from school or nursery?**

Base: All with children in school or nursery (VERSION A = 143; VERSION B = 136)

	Version A<br>%	Version B<br>%
Very likely	37	30
Fairly likely	18	16
Not very likely	31	34
Not at all likely	11	16
Don't know	3	4

Q25. **Please now think back to the situation I just described where there has been an outbreak of pneumonic plague in your area. If you had come into contact with one of the three people known to have pneumonic plague and were asked by doctors to take a course of antibiotics as a precautionary measure, how likely or unlikely is it that you would take them? The antibiotics would be provided for free.**

	Version A<br>%	Version B<br>%
Very likely	92	93
Fairly likely	5	5
Not very likely	1	1
Not at all likely	1	1
Don't know	*	*

Q26. **Why do you say you are unlikely to take the antibiotics?**
Base: All unlikely to take antibiotics (VERSION A = 11; VERSION B = 9)

	Version A<br>N	Version B<br>N
I would not be worried about catching plague	0	1
I am allergic to antibiotics	1	1
I would be worried about side effects of the antibiotics	0	1
I do not take medicines in general	1	3
I would not want to take antibiotics until I was sure I had the disease	7	2
I would be concerned about becoming immune to antibiotics	0	0
I would not want to pay for the antibiotics	0	0
Other	2	1

BASE = ALL (1,005)

Now carrying this hypothetical situation further, I would now like you to imagine that it is several days since the three people in your area have been diagnosed with pneumonic plague. The police have located the source of the plague outbreak. This was a canister of toxic material hidden at a busy train station. The police have confirmed this was left there deliberately by terrorists. Over a hundred people across the region have now been diagnosed with plague and a number of them have died.

Q27. **If this situation occurred, how worried, if at all, would you be about catching pneumonic plague yourself?**

Q28. **If this situation occurred, how worried, if at all, would you be about your close family or loved ones catching pneumonic plague?**

	Q27<br>%	Q28<br>%
Very worried	52	65
Fairly worried	31	23
Not very worried	12	8
Not at all worried	4	3
Don’t know	*	*

Q29.-Q36. **And how likely, if at all, would you be to do each of the following actions I am going to read out?**

		Very likely %	Fairly likely %	Not very likely %	Not at all likely %	Don't know %	Not applicable (Q32 ONLY) %
Q29.	**Make sure your home was stocked up with food and supplies**	59	20	14	7	*	N/A
Q30.	**Carry on as normal**	36	29	23	12	*	N/A
Q31.	**Leave the area**	11	12	37	40	1	N/A
Q32.	**Avoid going to work or college if applicable**	28	15	21	16	0	20
Q33.	**Avoid crowded areas such as public transport, supermarkets or pubs**	62	21	10	6	1	N/A
Q34.	**Avoid leaving your home if at all possible**	35	20	27	18	1	N/A
Q35.	**Seek medical advice**	59	20	13	8	1	N/A
Q36.	**Try to obtain a supply of antibiotics for yourself or your family/household**	56	15	17	10	1	N/A

Q37. **You said you would be likely to leave the area. Where do you think would you go to?**
Base: All likely to leave the area (228)

	%
Elsewhere in the UK, but within my region of the country	12
Elsewhere in the UK, outside my region of the country	56
Elsewhere in Europe but outside the UK	17
Outside of Europe	12
Don't know	3

Q38. **You said you would be likely to see medical advice. Where would you seek medical advice?**
Base: All likely to see medical advice (793)

	%
GP	80
NHS Direct	28
Internet	16
Hospital	16
Media like newspapers, television or radio	6
Pharmacy	5
Medically qualified friend or relative	5
Health clinic	1
Another friend or relative (not medically qualified)	1
Government	1
Police	*
Other	4
Don't know	*

Q39. **You said you would be likely to try to obtain a supply of antibiotics. Where would you try to obtain antibiotics from?**

Base: All likely to try to obtain antibiotics (793)

	%
GP	77
Pharmacy	24
Local hospital	21
Internet	6
NHS direct	3
Health centre	2
Medically qualified friend or relative	1
Hospital	1
Government	1
Black market	1
Another friend or relative (not medically qualified)	1
Police	*
Other	3
Don’t know	2

Q40. **How likely or unlikely is it that you would keep your children home from school or nursery?**

Base: All with children in school or nursery (329)

	%
Very likely	47
Fairly likely	15
Not very likely	14
Not at all likely	7
Don’t know	2

SPLIT SAMPLE: VERSION A AND VERSION B.

VERSION A (n = 492)

This is the final element to the hypothetical situation: Health officials are saying that anyone who was at the train station where the canister of toxic material was found should go to a local mass treatment centre. When people arrive at the centre, they will be assessed by a team of specialists and may be given antibiotics as a precautionary measure.

Q41. **Assuming that you had been to the affected train station, which of the following would you do first?**

	%
Go to the treatment centre as soon as possible	75
Visit your GP	11
Speak to NHS Direct (CLARIFY IF NECESSARY: the NHS’ telephone advice service)	6
Go to the treatment centre at some point when it was convenient, but not immediately	4
Go to your local hospital	2
Look for more information elsewhere (ALWAYS LAST)	3
Do nothing	*
Don’t know	*

Q42. **Why would you <u>not</u> go the treatment centre as soon as possible?**
Base: All who would not go to the centre as soon as possible (125)

	%
I would be worried about catching plague at the treatment centre	37
I would want more information first	15
I would prefer to obtain medical advice elsewhere	11
Too crowded/Busy	9
Depends on distance/Which was closer	6
Wouldn't be worried about it	4
I would not want to leave my house or risk any type of travel	4
Wouldn't want to infect anybody else	3
Prefer to see my GP/Trust my GP	2
Mobility problems/I have problems getting around	2
Would need to be at work	1
I am responsible for children or other dependents, and would not want to bring them along to the centre in case they caught the disease	1
I would not want to take antibiotics	1
I would be worried about not being allowed to leave the treatment centre	1
I would not believe the specialist medical staff at the centre would be expert enough	0
Other	9
Don't know	7

Q43. **And again assuming you had been to the affected train station, and now assuming you had soon after developed flu-like symptoms, which if any of the following would you do <u>first</u>?**

	%
Go to the treatment centre as soon as possible	66
Visit your GP	15
Speak to NHS Direct (CLARIFY IF NECESSARY: the NHS' telephone advice service)	9
Go to your local hospital	7
Go to the treatment centre at some point when it was convenient, but not immediately	1
Other	*
Look for more information elsewhere (ALWAYS LAST)	*
Do nothing	*
None of these	*
Don't know	1

Q44. **And if you had <u>not</u> been to the affected train station, how likely would you be to go to the treatment centre anyway?**

	%
Very likely	9
Fairly likely	9
Not very likely	46
Not at all likely	36
Don't know	*

Q45. **Why would you go to the treatment centre?**

Base: All who would go to the treatment centre (88)

	%
To get a medical check-up	55
To obtain information	25
To obtain antibiotics	12
As a precaution/To be on the safe side/Reassurance	9
To take a household member, child or dependent if they had been exposed	3
Other	6
Don't know	1

Q46. **And finally, if you had not been to the affected train station, but had developed flu-like symptoms, which of the following would you do first?**

	%
Visit your GP	40
Go to the treatment centre as soon as possible	29
Speak to NHS Direct (CLARIFY IF NECESSARY: the NHS' telephone advice service)	20
Go to your local hospital	5
Look for more information elsewhere	3
Go to the treatment centre at some point when it was convenient, but not immediately	2
Do nothing	1
Other	*

VERSION B (n = 513)

If somebody had been at the train station, health officials would ask them to stay at home for up to 7 days. They would be asked to monitor themselves for any signs of illness, to take their temperature regularly and to call a special phone number if they developed a high temperature or any flu-like symptoms. People asked to stay at home would not be allowed to come into direct contact with anybody from outside their home.

Q41. **From a practical standpoint, how easy or difficult for you would it be for you to keep yourself at home for seven days and not leave the house? You would be allowed to interact with family and household members living with you, but you would not be allowed to leave your home.**

	%
Very easy	43
Fairly easy	31
Fairly difficult	13
Very difficult	12
Don't know	1

Q42. **If you had been at the affected train station, how likely or unlikely is it that you would actually keep yourself at home for seven days?**

	%
Very likely	70
Fairly likely	22
Not very likely	5
Not at all likely	3
Don't know	1

Q43. **Please imagine that you have been asked to stay at home because you had been exposed to the disease. I am going to read out some things that might persuade you to stay in your home for seven days.**

Please tell me which two or three, if any, of these would be the <u>most</u> likely to persuade you to stay indoors. Please assume that you are provided with food and water at your home for the duration of the seven days.

	%
**Being telephoned daily by a medical expert who checked on your health**	56
**Being fully compensated for any financial losses as a result of staying at home**	39
**A trusted friend or relative, or a social worker if you prefer, looking after your children or other dependents, so that they did not have to stay at home with you**	30
**Being provided with a mobile phone with internet access with which to make free telephone calls and go online while at home**	27
**If you could be prosecuted for not staying at home**	24
**Your employer or college helping you to work from home, if applicable**	20
**Being provided with free computer games, books or other activities to help pass the time while you were at home**	17
None of these	6
Don’t know	1

DEMOGRAPHICS

Based on All respondents (1,005)

QD1. **Is the home you are living in …?** READ OUT

		%
A	Being bought on a mortgage	41
B	Owned outright	34
C	Rented (private)	9
D	Rented (Local Authority/Council)	8
E	Rented (Housing association/Trust)	5
	Other	1
	Refused	2

QD2. **And is your dwelling…?** PROMPT AS NECESSARY

	%
Detached villa	24
Semi-detached villa	30
Bungalow	6
Semi-detached bungalow	3
Terraced house	21
Four-in-a-block	2
Tenement flat	3
Multi-storey flat	2
Maisonette	1
Modern apartment/loft apartment/studio / other flat	2
Other	4
Don't know	1

QD3. **Which of the following ethnic backgrounds describes you the best?**

	%
White British	88
Irish	2
Other	3
Mixed White and black Caribbean	1
White and Asian	*
Other	1
Black or black British -	
Caribbean	1
African	1
Other	0
Asian or Asian British Indian	1
Pakistani	1
Bangladeshi	0
Other	1
Chinese or ethnic group – Chinese	*
Other ethnic background	*
Refused	1

QD4. **Now I would like to ask you a question about faith and religion. What is your religion, if any?** IF ANSWER 'CHRISTIAN' PLEASE ASK QD4B. SINGLE CODE ONLY QD4B) ASK IF CHRISTIAN **What denomination are you?** SINGLE CODE ONLY

	%
Christian	60
Muslim	1
Hindu	1
Sikh	*
Orthodox Greek/Russian	0
Buddhist	*
Jewish	*
Other	4
None	31
Don't know	*
Refused	2

QD4B **What denomination are you?** SINGLE CODE ONLY

Base: All who say they are Christian (606)

	%
Church of England	66
Roman Catholic	16
Church of Scotland	5
Free Church/Non-Conformist (Methodist, Baptist etc)	9
Other Protestant	4

QD5. **Into which of the following categories would you place your annual total household income from all sources before tax and any other deductions?** READ OUT. SINGLE CODE ONLY

	%
Under £10,000	13
Over £10,000 but less than £20,000	18
Over £20,000 but less than £30,000	19
Over £30,000 but less than £40,000	13
Over £40,000	24
Refused	8
Don't know	6

IF REFUSE – PLEASE ADD BANDS

QD6. **Gender**

	%
Male	48
Female	52

QD7. **What was your age at your last birthday, if I may ask?** INTERVIEWER: CODE EXACT AGE.

	%
16-24	14
25-34	16
35-44	19
45-54	16
55-64	14
65+	20

QD8. **And are you, yourself…?** PROMPT AS NECESSARY. SINGLE CODE

	%
Working full time (30hrs/wk+)	45
Working part time (8–29 hrs/wk)	14
Not working (ie under 8 hrs) – housewife	4
Not working (ie under 8 hrs) – unemployed (registered)	1
Not working (ie under 8 hrs) – unemployed (not registered but looking for work)	1
Not working (ie under 8 hrs) – retired	24
Not working (ie under 8 hrs) – student	6
Not working (ie under 8 hrs) – other (inc. disabled)	4
Don't know	*
Refused	*

QD9. **How many years of full-time education have you had?**

	%
1-9	6
10-12	35
13-15	27
16-18	23
19+	7
Don't know	1
Refused	1

QD10. **Including yourself, how many people do you live with?**

	%
1	29
2	49
3	14
4	8
5	2
6+	1
Refused	0

QD11. **How many cars or light vans are there in your household?** SINGLE CODE ONLY

	%
1 car or light van	41
2 cars or light vans	33
3+ cars or light vans	9
None	16
Refused	1

QD12. **Do you have access to the internet at home?**

	%
Yes	76
No	23
Refused	1
