# Supplementary material for: Perceptions and Reactions with Regard to Pneumonic Plague
Source: Emerg Infect Dis. 2010 Jan;16(1):120–2. doi: 10.3201/eid1601.081604 (PMC2874346; doi:10.3201/eid1601.081604)
Supplement: Technical Appendix 2 — Perceptions and Reactions with Regard to Pneumonic Plague [file 08-1604_Techapp2-s2.pdf]

Perceptions and Reactions with Regard to Pneumonic Plague

Technical Appendix 2

Table 1. Odds ratios (95% confidence interval) between demographic variables and precautionary behavioral responses to a hypothetical pneumonic plague outbreak affecting three people (stage 2); significant results ($p<0.05$) are in **boldface**

		**Stocking up on food**	**Leaving the area**	**Avoiding others**	**Seeking medical advice**	**Trying to obtain antibiotics**
	**Number (%) reporting being "very" or "fairly" likely to perform that behaviour**	673 (67.2)	132 (13.3)	746 (74.2)	667 (66.4)	591 (59.4)
**Predictor variable**	**Variable levels (n)**					
Home ownership	Non-owner (228)	1.0 (0.7 to 1.4)	**1.9 (1.2 to 2.8)**	0.7 (0.5 to 1.0)	**1.4 (1.0 to 2.0)**	**1.4 (1.0 to 1.9)**
	Owner (752)	Reference	Reference	Reference	Reference	Reference
Ethnicity	Non-white (69)	1.3 (0.7 to 2.2)	1.8 (1.0 to 3.3)	1.1 (0.6 to 1.9)	**2.1 (1.1 to 3.8)**	**2.2 (1.3 to 3.9)**
	White (936)	Reference	Reference	Reference	Reference	Reference
Sex	Female (562)	**1.5 (1.2 to 2.0)**	1.0 (0.7 to 1.5)	1.0 (0.8 to 1.4)	1.1 (0.9 to 1.5)	1.1 (0.9 to 1.5)
	Male (443)	Reference	Reference	Reference	Reference	Reference
Age	16 to 34 (265)	**0.7 (0.5 to 1.0)**	**2.1 (1.3 to 3.3)**	**0.6 (0.4 to 0.9)**	0.8 (0.6 to 1.2)	0.8 (0.5 to 1.0)
	35 to 54 (376)	**0.7 (0.5 to 0.9)**	1.3 (0.8 to 2.0)	0.8 (0.6 to 1.1)	0.9 (0.6 to 1.2)	0.8 (0.6 to 1.0)
	55 or over (364)	Reference	Reference	Reference	Reference	Reference
Working status	Not in work (397)	**1.6 (1.2 to 2.2)**	1.4 (0.9 to 2.0)	**1.7 (1.2 to 2.2)**	**1.5 (1.1 to 2.0)**	**1.4 (1.1 to 1.8)**
	Working (602)	Reference	Reference	Reference	Reference	Reference
Number of	1 to 12 years (416)	**1.6 (1.1 to 2.1)**	**1.7 (1.1 to 2.7)**	0.9 (0.7 to 1.3)	**2.2 (1.6 to 3.0)**	**2.2 (1.6 to 3.0)**

years in education						
	13 to 15 years (259)	**2.0 (1.4 to 2.8)**	1.4 (0.8 to 2.4)	1.0 (0.7 to 1.5)	**1.7 (1.2 to 2.4)**	**1.7 (1.2 to 2.4)**
	16 or more (307)	Reference	Reference	Reference	Reference	Reference
Social grade	C2DE (427)	**1.6 (1.2 to 2.1)**	**1.7 (1.2 to 2.5)**	1.1 (0.8 to 1.5)	**1.8 (1.4 to 2.4)**	**1.9 (1.5 to 2.5)**
	ABC1 (578)	Reference	Reference	Reference	Reference	Reference
Number of people living at home	1 (243)	1.2 (0.9 to 1.7)	0.9 (0.6 to 1.4)	0.9 (0.6 to 1.2)	0.8 (0.6 to 1.1)	0.9 (0.6 to 1.2)
	2 (332)	1.1 (0.8 to 1.5)	0.5 (0.3 to 0.8)	1.1 (0.8 to 1.5)	0.9 (0.7 to 1.2)	0.9 (0.7 to 1.2)
	3 or more (424)	Reference	Reference	Reference	Reference	Reference
Parental status	Children at home (336)	1.0 (0.8 to 1.3)	1.2 (0.8 to 1.8)	1.1 (0.8 to 1.4)	1.3 (1.0 to 1.7)	1.1 (0.8 to 1.4)
	No children at home (668)	Reference	Reference	Reference	Reference	Reference
Long standing illness or disability	Illness present (246)	1.1 (0.8 to 1.6)	1.1 (0.8 to 1.7)	1.0 (0.8 to 1.5)	1.4 (1.0 to 1.9)	1.1 (0.8 to 1.5)
	No illness (757)	Reference	Reference	Reference	Reference	Reference

Publisher: CDC; Journal: Emerging Infectious Diseases
Article Type: Dispatch; Volume: ; Issue: ; Year: ; Article ID: 08-1604
DOI: ; TOC Head:

Table 2. Odds ratios (95% confidence intervals) between demographic variables and precautionary behavioral responses to a hypothetical pneumonic plague outbreak affecting 100 people (stage 3); significant results (p<0.05) are in **boldface**

		**Stocking up on food**	**Leaving the area**	**Avoiding others**	**Seeking medical advice**	**Trying to obtain antibiotics**
	**Number (%) reporting being "very" or "fairly" likely to perform that behaviour**	798 (79.8%)	223 (22.4%)	850 (84.6%)	792 (79.4%)	724 (72.5%)
**Predictor variable**	**Variable levels (n)**					
Home ownership	Non-owner (228)	0.9 (0.6 to 1.3)	**1.8 (1.3 to 2.6)**	0.9 (0.6 to 1.3)	**1.6 (1.1 to 2.4)**	1.4 (1.0 to 2.0)
	Owner (752)	Reference	Reference	Reference	Reference	Reference
Ethnicity	Other (69)	1.1 (0.6 to 2.0)	**1.8 (1.1 to 3.1)**	1.1 (0.5 to 2.2)	1.2 (0.7 to 2.4)	1.9 (1.0 to 3.5)
	White (936)	Reference	Reference	Reference	Reference	Reference
Sex	Female (562)	**2.1 (1.5 to 2.9)**	**1.5 (1.1 to 2.0)**	1.3 (0.9 to 1.8)	**1.7 (1.2 to 2.3)**	**1.5 (1.1 to 2.0)**
	Male (443)	Reference	Reference	Reference	Reference	Reference
Age	16 to 34 (265)	1.0 (0.7 to 1.5)	**2.9 (1.9 to 4.2)**	1.2 (0.8 to 1.8)	**1.6 (1.1 to 2.4)**	1.0 (0.7 to 1.5)
	35 to 54 (376)	1.0 (0.7 to 1.5)	**2.0 (1.3 to 2.9)**	1.2 (0.8 to 1.7)	1.2 (0.8 to 1.7)	0.9 (0.7 to 1.3)
	55 or over (364)	Reference	Reference	Reference	Reference	Reference
Working status	Not in work (397)	**1.7 (1.2 to 2.3)**	1.1 (0.8 to 1.4)	1.1 (0.8 to 1.6)	1.2 (0.9 to 1.7)	**1.4 (1.1 to 1.9)**
	Working (602)	Reference	Reference	Reference	Reference	Reference
Number of years in education	1 to 12 years (416)	1.4 (1.0 to 2.0)	1.0 (0.7 to 1.5)	0.7 (0.5 to 1.1)	**2.0 (1.4 to 2.9)**	**2.1 (1.5 to 3.0)**
	13 to 15 years (259)	1.5 (1.0 to 2.3)	1.0 (0.7 to 1.5)	1.0 (0.6 to 1.6)	**1.8 (1.2 to 2.7)**	**1.7 (1.2 to 2.5)**
	16 or more (307)	Reference	Reference	Reference	Reference	Reference
Social grade	C2DE (427)	1.4 (1.0 to 1.9)	1.2 (0.9 to 1.6)	1.2 (0.8 to 1.7)	**1.5 (1.1 to 2.1)**	**1.6 (1.2 to 2.2)**
	ABC1 (578)	Reference	Reference	Reference	Reference	Reference
Number of people living at home	1 (243)	1.1 (0.8 to 1.7)	**0.6 (0.5 to 0.9)**	0.7 (0.5 to 1.1)	0.7 (0.5 to 1.1)	0.8 (0.6 to 1.2)
	2 (332)	1.2 (0.8 to 1.7)	**0.4 (0.3 to 0.6)**	0.8 (0.5 to 1.2)	0.8 (0.6 to 1.2)	0.9 (0.6 to 1.2)

	3 or more (424)	Reference	Reference	Reference	Reference	Reference
Parental status	Children at home (336)	1.1 (0.8 to 1.6)	**1.6 (1.2 to 2.2)**	1.4 (0.9 to 2.0)	**1.7 (1.2 to 2.5)**	1.2 (0.9 to 1.6)
	No children at home (668)	Reference	Reference	Reference	Reference	Reference
Long standing illness or disability	Illness present (246)	1.3 (0.9 to 1.9)	1.0 (0.7 to 1.4)	1.0 (0.7 to 1.6)	1.3 (0.9 to 1.9)	1.3 (0.9 to 1.8)
	No illness (757)	Reference	Reference	Reference	Reference	Reference

Publisher: CDC; Journal: Emerging Infectious Diseases
Article Type: Dispatch; Volume: ; Issue: ; Year: ; Article ID: 08-1604
DOI: ; TOC Head:

Table 3. Odds ratios (95% confidence interval) between demographic variables and likely compliance with official recommendations during a hypothetical pneumonic plague outbreak; significant results (p<0.05) are in **boldface**

	**Recommended action:**	**Attending mass treatment centre if potentially at risk**	**Not attending mass treatment centre if not potentially at risk**	**Remaining indoors at home for 7 days if requested**
	**Number (%) reporting being unlikely to comply with the recommended behaviour**	107 (21.3%)	88 (17.6%)	40 (8%)
**Predictor variable**	**Variable levels**			
Home ownership	Non-owner	0.7 (0.4 to 1.2)	**2.1 (1.2 to 3.5)**	1.6 (0.8 to 3.2)
	Owner	Reference	Reference	Reference
Ethnicity	Non-white	**2.3 (1.0 to 4.9)**	1.5 (0.6 to 3.5)	1.8 (0.7 to 5.0)
	White	Reference	Reference	Reference
Sex	Female	0.7 (0.5 to 1.1)	1.5 (1.0 to 2.5)	**0.4 (0.2 to 0.8)**
	Male	Reference	Reference	Reference
Age	16 to 34	**0.4 (0.2 to 0.7)**	1.5 (0.8 to 2.7)	1.0 (0.4 to 2.2)
	35 to 54	**0.6 (0.3 to 0.9)**	0.9 (0.5 to 1.6)	1.2 (0.6 to 2.5)
	55 or over	Reference	Reference	Reference
Working status	Not in work	**2.3 (1.5 to 3.5)**	1.1 (0.7 to 1.8)	0.9 (0.4 to 1.7)
	Working	Reference	Reference	Reference
Number of years in education	1 to 12 years	1.1 (0.6 to 1.7)	**2.0 (1.1 to 3.7)**	1.0 (0.4 to 2.2)
	13 to 15 years	0.7 (0.4 to 1.3)	1.7 (0.9 to 3.3)	1.3 (0.6 to 3.1)
	16 or more	Reference	Reference	Reference
Social grade	C2DE	**1.5 (1.0 to 2.4)**	**2.0 (1.3 to 3.3)**	1.2 (0.6 to 2.2)
	ABC1	Reference	Reference	Reference
Number of people living at home	1	**2.3 (1.4 to 4.0)**	1.2 (0.7 to 2.2)	0.6 (0.3 to 1.5)
	2	1.4 (0.8 to 2.4)	1.2 (0.7 to 2.1)	0.8 (0.4 to 1.4)
	3 or more	Reference	Reference	Reference
Parental status	Children at home	**0.6 (0.3 to 0.9)**	1.0 (0.6 to 1.6)	1.1 (0.5 to 2.1)
	No children at home	Reference	Reference	Reference

Long standing illness or disability	Illness present	1.2 (0.7 to 1.9)	0.8 (0.5 to 1.4)	1.4 (0.7 to 2.9)
	No illness	Reference	Reference	Reference

Table 4. Adjusted odds ratios (95% confidence interval) between perceptions relating to pneumonic plague and compliance with official recommendations during a hypothetical pneumonic plague outbreak. All odds ratios adjust for home ownership, ethnicity, sex, age, working status, number of years in education ,social grade, number of people living at home, and parental status; significant results ($p<0.05$) are in **boldface**

	**Recommended action:**	**Attending mass treatment centre if potentially at risk**	**Not attending mass treatment centre if not potentially at risk**	**Remaining indoors at home for 7 days if requested**
	**Number (%) reporting being unlikely to comply with the recommended behaviour**	107 (21.3%)	88 (17.6%)	40 (8%)
**Predictor variable**	**Variable levels**			
If someone catches pneumonic plague they would feel unwell within 24hrs	Very or fairly likely	2.0 (0.9 to 4.2)	1.4 (0.7 to 2.7)	1.0 (0.4 to 2.8)
	Not very or not at all likely	Reference	Reference	Reference
There have been cases of pneumonic plague in Britain in the past 10 years	Very or fairly likely	1.0 (0.5 to 1.8)	**2.3 (1.3 to 4.0)**	0.8 (0.3 to 1.9)
	Not very or not at all likely	Reference	Reference	Reference
If you come within 6 feet of someone who had pneumonic plague and was clearly ill, you would probably catch the disease	Very or fairly likely	1.0 (0.6 to 1.8)	1.0 (0.5 to 1.8)	1.3 (0.6 to 2.9)
	Not very / not at all likely	Reference	Reference	Reference
If you come within 6 feet of someone who had pneumonic plague but who had not yet developed any signs of illness, you would probably catch the disease	Very or fairly likely	0.8 (0.5 to 1.4)	**2.8 (1.5 to 5.3)**	0.7 (0.3 to 1.4)
	Not very or not at all likely	Reference	Reference	Reference
Unless they receive immediate treatment, then most people who catch pneumonic plague will die from it	Very or fairly likely	0.7 (0.4 to 1.4)	1.7 (0.8 to 3.9)	1.1 (0.4 to 2.7)

	Not very or not at all likely	Reference	Reference	Reference
If antibiotics are administered immediately after a person has been infected, they would probably survive	Very or fairly likely	1.0 (0.4 to 2.5)	**0.3 (0.2 to 0.6)**	0.6 (0.2 to 2.1)
	Not very or not at all likely	Reference	Reference	Reference
If someone with plague has been in a room, how long would it take after they leave before it is safe to enter the room	Less than 1 day	0.8 (0.4 to 1.6)	0.7 (0.4 to 1.4)	1.1 (0.4 to 2.8)
	1 to 2 days	1.0 (0.5 to 1.9)	0.8 (0.4 to 1.5)	1.3 (0.5 to 3.5)
	3 days or more	Reference	Reference	Reference
